# Supplementary material for: Apicobasal Surfaceome Architecture Encodes for Polarized Epithelial Functionality and Depends on Tumor Suppressor PTEN
Source: Int J Mol Sci. 2022 Dec 19;23(24):16193. doi: 10.3390/ijms232416193 (PMC9788433; doi:10.3390/ijms232416193)
Supplement: Supplementary file 1 [file ijms-23-16193-s001.zip › ijms-2026488-supplementary.pdf]

Supplemental Material

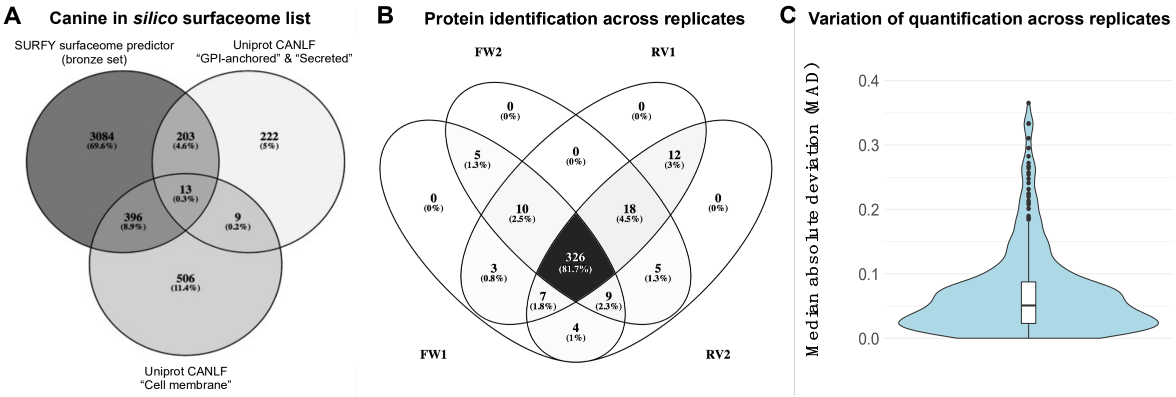

Figure S1: Protein filtering, identification and quantification across replicates using SILAC-complemented chemoproteomics to map polarized protein distributions across the apicobasal surfaceome. **(A)** Assembly of a canine *in silico* surfaceome list for data filtering using UniprotKB entries annotated as "Cell membrane", "GPI-anchor", and "secreted" for *Canis lupus familiaris* (CANLF), as well as the predicted surface proteins contained in the bronze set of "Surfy" (Bausch-Fluck et al. 2018). **(B)** Overlap of identified proteins across replicates and **(C)** a low median absolute deviation (with a median of 5%) shows high reproducibility of the apicobasal protein quantification based on SILAC-complemented chemoproteomics.

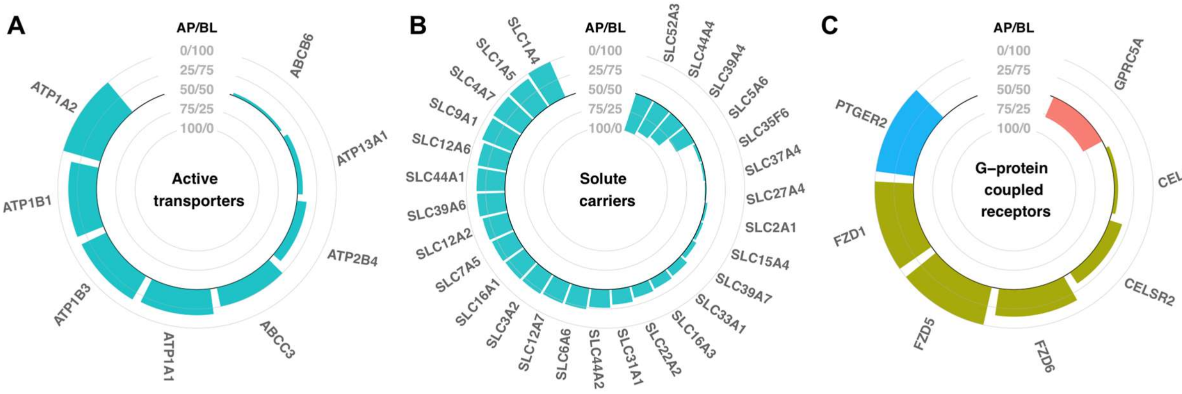

Figure S2: Quantitative apicobasal distribution (AP/BL ratio) of selected protein classes on filter-grown MDCK cells based on SILAC-complemented chemoproteomics: **(A)** active transporter proteins, **(B)** solute carrier proteins, **(C)** G-protein coupled receptor proteins. Classification according to the Gene Ontology and KEGG pathway databases (n=4, with 2 SILAC forward and 2 reverse experiments).

Table S1: List of all cell surface proteins quantified across the apicobasal surfaceome of filter-grown MDCK cells based on SILAC-complemented chemoproteomics. Apicobasal protein distribution is presented as apical fraction (AP) of the apicobasal protein pool [AP/(AP+BL)] across 4 replicates (2 fw=SILAC forward; 2 rv=SILAC reverse experiments), where "NaN" represents non-detected/quantified proteins in a specific replicate and variation is given as median absolute deviation (MAD). Colour-scale indicates descending apical fraction from red to green.

Gene name	Uniprot ID	AP Median	AP fw1	AP fw2	AP rv1	AP rv2	MAD
ABCB6	F1PRH1	0.465	0.377	0.462	0.469	0.495	0.031
ABCC3	J9NY13	0.252	0.110	0.243	0.261	0.260	0.042
ACAT1	F1PC58	0.568	0.563	0.582	0.505	0.573	0.022
ACE2	J9P7Y2	0.908	0.901	0.889	0.915	0.940	0.016
ACSF2	F1PTR3	0.470	0.521	0.477	0.436	0.463	0.025
ADAM10	F1PGR0	0.244	0.246	0.236	0.243	0.255	0.005
ADAM17	F1PFZ9	0.291	0.361	0.294	0.281	0.287	0.022
ADAM28	F1PRX8	0.363	0.000	0.346	0.379	0.430	0.116
ADAM9	J9P4T2	0.389	0.409	0.417	0.352	0.370	0.026
AGPAT1	E2RCA0	0.497	0.519	0.495	0.467	0.498	0.014
AGRN	F1Q2Z6	0.257	0.204	0.178	0.310	0.382	0.078
ANO6	J9NZU1	0.193	0.112	0.174	0.236	0.212	0.040
ANXA2	Q6TEQ7	0.535	0.575	0.577	0.446	0.496	0.052
APMAP	E2RPE9	0.635	0.478	0.580	0.694	0.690	0.081
ASAH1	F1PWA9	0.557	0.416	0.557	NaN	0.664	0.082
ATP13A1	F1PRS5	0.432	0.560	0.000	NaN	0.432	0.187
ATP1A1	J9NZA9	0.169	0.172	0.167	0.171	0.166	0.002
ATP1A2	F1PL53	0.000	0.000	0.000	0.195	NaN	0.065
ATP1B1	P06583	0.136	0.148	0.112	0.124	0.150	0.016
ATP1B3	F1Q0S2	0.146	0.136	0.156	0.110	0.216	0.032
ATP2B4	F1PHQ7	0.375	0.524	0.427	0.231	0.322	0.100
B2M	E2RN10	0.221	0.234	0.259	0.208	0.205	0.020
BCAM	J9P3V6	0.213	0.213	0.204	0.212	0.219	0.004
BCHE	F1PEN8	0.376	0.548	NaN	NaN	0.204	0.172
BMPR1A	E2QW66	0.000	NaN	NaN	0.000	0.000	0.000
BMPR2	F1PCC1	0.000	NaN	NaN	0.000	0.000	0.000
BSG	E2QZT4	0.130	0.170	0.144	0.092	0.116	0.027
BST1	E2RJT2	0.908	0.904	0.898	0.912	0.958	0.017
BST2	J9NVI2	0.652	0.711	0.689	0.545	0.614	0.060
CA12	F1Q440	0.243	0.220	0.141	0.266	0.338	0.061
CA14	F1P7K9	0.369	0.383	0.369	0.369	0.256	0.032
CADM1	F1P6Z9	0.203	0.202	0.204	0.189	0.234	0.012
CALU	E2RN38	0.894	0.474	1.000	1.000	0.788	0.184
CANX	P24643	0.535	0.543	0.645	0.526	0.513	0.037
CAV1	P33724	0.538	0.600	0.564	0.490	0.512	0.041
CAV2	O46550	0.529	0.510	0.513	0.563	0.545	0.021
CD109	F1PM26	0.586	0.451	0.393	0.757	0.721	0.158
CD14 (607076)	A0A1S7J0A8	1.000	NaN	NaN	1.000	1.000	0.000
CD151	J9P4E7	0.244	0.311	0.252	0.236	0.231	0.024
CD276	F1PDP5	0.213	0.212	0.241	0.213	0.210	0.008
CD302	F1PGD5	0.499	0.502	0.488	0.497	0.530	0.012
CD40	G1K297	0.256	0.316	0.312	0.179	0.199	0.062
CD44	J9P423	0.217	0.213	0.214	0.220	0.265	0.014
CD47	J9P735	0.581	0.545	0.596	0.566	1.000	0.121
CD55	F1PCI2	0.125	NaN	NaN	0.249	0.000	0.125
CD58	F1PBA0	0.097	0.047	0.074	0.121	0.135	0.034
CD59	J9P4N4	0.732	0.738	0.773	0.697	0.726	0.022
CD63	F1PIT4	0.507	0.699	0.718	0.314	0.250	0.213
CD74	E2RF90	0.334	0.257	0.328	0.340	0.345	0.025
CD81	F1PB66	0.027	0.000	0.000	0.055	0.106	0.040
CD82	J9P9V8	0.353	0.000	0.484	0.293	0.413	0.151
CD9	F1PQM7	0.342	0.400	0.362	0.298	0.322	0.035

CD99	J9PA14	0.258	0.253	0.263	0.267	0.250	0.007
CDC42	P60952	0.456	0.180	0.265	0.763	0.647	0.241
CDCP1	F1PV63	0.357	0.333	0.307	0.381	0.426	0.042
CDH1	F1PAA4	0.196	0.195	0.171	0.219	0.197	0.013
CDH10	E2R858	0.231	0.000	0.224	0.239	0.319	0.083
CDH16	E2REV2	0.176	0.201	0.176	0.167	0.177	0.009
CDH17	J9NTL4	0.521	0.469	0.457	0.573	0.576	0.056
CDH3	F1PAA3	0.098	0.000	0.000	0.197	0.355	0.138
CDH6	E2R0Z0	0.418	0.413	0.402	0.428	0.424	0.009
CEACAM1	Q004B2	0.956	0.904	0.965	0.988	0.947	0.025
CELSR1	F1PLY1	0.453	0.548	0.450	0.456	0.415	0.035
CELSR2	E2R4F0	0.329	0.350	0.331	0.286	0.327	0.017
CENPV	F1PH09	0.519	0.382	0.425	0.629	0.612	0.108
CEPT1	E2QV13	1.000	1.000	NaN	0.459	1.000	0.180
CLDN1	J9P0K9	0.424	0.458	0.460	0.000	0.390	0.132
CLDN2	Q95KM6	0.400	0.483	0.446	0.301	0.353	0.069
CLDN3	Q95KM5	0.371	0.429	0.456	0.286	0.314	0.071
CLDN4	E2RNC2	0.217	0.184	0.294	0.250	0.041	0.080
CLDN7	E2R5S3	0.101	0.202	0.000	NaN	NaN	0.101
CLDND1	F1PYH4	0.063	0.125	0.000	NaN	NaN	0.063
CLEC7A	E2RFU8	0.385	0.379	0.390	0.340	0.399	0.018
CLU	P25473	0.639	0.640	0.600	0.654	0.639	0.014
CNNM3	E2RGW9	0.000	0.000	0.000	NaN	0.000	0.000
CNNM4	F1PJ05	0.143	0.189	0.140	0.118	0.145	0.019
COL18A1	F6Y3U7	0.176	0.105	0.084	0.344	0.247	0.101
COL4A1	Q28271	0.424	0.266	0.237	0.618	0.581	0.174
COL4A2	F1Q129	0.366	0.172	0.077	0.718	0.560	0.257
CP	F1PAX2	0.839	0.824	0.842	0.837	0.851	0.008
CPM	F1PGL5	0.634	0.661	0.669	0.566	0.608	0.039
CRELD1	F1PD72	0.613	0.630	0.596	0.732	0.535	0.058
CRIM1	E2R003	0.000	0.000	0.000	0.000	0.285	0.071
CS	F1PV92	0.487	0.551	0.539	0.402	0.436	0.063
CTNNB1	B6V8E6	0.453	0.515	0.317	0.490	0.416	0.068
CTSA	F1P9I9	0.504	0.362	0.428	0.580	0.582	0.093
CTSB	E2R6Q7	0.464	0.506	0.357	0.426	0.502	0.056
CTSD	Q4LAL9	0.458	0.479	0.499	0.378	0.437	0.041
CTSH	F6X9C1	0.187	NaN	0.000	NaN	0.374	0.187
CTSS	Q8HY81	0.489	NaN	0.000	0.489	0.556	0.185
CTSZ	F1PIF2	0.433	0.000	0.530	0.407	0.459	0.145
CXADR	F1P992	0.296	0.325	0.289	0.266	0.302	0.018
CYB5R1	E2RF18	0.530	0.663	0.565	0.416	0.496	0.079
CYP20A1	F1PLQ3	0.495	0.493	0.490	0.498	0.545	0.015
DAG1	Q9TSZ6	0.375	0.340	0.325	0.503	0.411	0.062
DAO	E2QV61	0.580	0.539	0.696	0.618	0.542	0.058
DCBLD2	F1PVX2	0.451	0.443	0.436	0.472	0.459	0.013
DDO	F1PAL6	0.512	0.531	0.599	0.426	0.493	0.053
DDR1	F1PGJ7	0.289	0.278	0.296	0.282	0.318	0.013
DDX17	F1PID8	0.517	0.445	0.504	0.543	0.530	0.031
DHCR7	E2RNQ5	0.497	0.564	0.568	0.351	0.431	0.088
dhrs4	Q0E9S7	0.556	0.385	0.567	0.546	0.566	0.050
DNAJC10	E2RCY4	0.484	0.000	0.546	0.496	0.471	0.143
DNASE1L1	F1PWT3	0.670	0.684	0.694	0.592	0.655	0.033
DPEP1	E2R4I6	1.000	NaN	1.000	1.000	1.000	0.000
DSC2	F1PEF1	0.251	0.251	0.246	0.252	0.256	0.003
DSC3	F1PEF0	0.402	0.391	0.425	0.413	0.376	0.018
DSG2	F1PGX2	0.476	0.518	0.502	0.450	0.438	0.033
ECE1	F1PK66	0.000	0.000	0.000	0.203	0.000	0.051
EFNA1	E2QUE4	0.177	0.191	0.000	0.296	0.163	0.081
EFNB1	E2QSB5	0.434	0.382	0.397	0.486	0.472	0.045
EGF	J9NZ75	0.533	0.553	0.792	0.506	0.512	0.082
EGFR	F1PF03	0.401	0.417	0.386	0.399	0.403	0.009
ELOVL1	E2RGY9	0.533	0.553	0.514	0.563	0.464	0.035
EMC1	F1PN78	0.545	NaN	0.618	0.545	0.219	0.133
ENPEP	F6XRM5	1.000	1.000	1.000	1.000	1.000	0.000
ENPP6	F1PZF9	1.000	1.000	1.000	1.000	0.835	0.041
ENTPD3	E2RE57	0.826	0.819	0.815	0.834	0.864	0.016

EPCAM	F1PM51	0.177	0.164	0.165	0.229	0.189	0.022
EPHA1	E2R6A6	0.147	0.085	0.112	0.183	0.193	0.045
EPHA2	F1Q4C5	0.345	0.338	0.403	0.348	0.342	0.018
EPHB2	F1PAF1	0.357	0.380	0.329	0.406	0.333	0.031
EPHB4	F1PRQ8	0.358	0.383	0.357	0.358	0.308	0.019
ERBB2	F1PIQ9	0.255	0.256	0.236	0.253	0.267	0.009
ERBB3	F1PVC5	0.112	0.000	0.064	0.179	0.159	0.068
ERBB4	F1PQ05	0.289	0.091	0.313	0.581	0.265	0.134
ERGIC3	E2QW07	0.644	NaN	0.644	1.000	0.000	0.333
ERMP1	F1PSG1	0.505	0.521	0.489	0.545	0.433	0.036
ERP29	J9P4L2	0.582	0.621	0.722	0.542	0.544	0.064
ESRP1	F1PSN8	0.383	0.383	0.511	0.303	NaN	0.069
F11R	F1PP71	0.221	0.209	0.206	0.244	0.234	0.016
FAM171A2	J9P3H5	0.182	0.233	0.073	0.131	0.272	0.075
FAS	F1P7Q7	0.209	0.285	0.133	0.063	0.328	0.104
FCGRT	E2R0L6	0.000	0.000	0.000	0.000	0.000	0.000
FGFR2	F1PPD8	0.270	0.293	0.127	0.264	0.276	0.045
FGFR3	F1PK02	0.429	0.520	0.569	0.338	0.291	0.115
FH	E2RGR9	0.499	0.563	0.531	0.402	0.468	0.056
FKBP10	E2QZA8	0.746	NaN	NaN	1.000	0.492	0.254
FLNA	F1PWW0	0.427	0.283	0.376	0.537	0.478	0.089
FLRT2	E2RB35	0.346	0.318	0.210	0.374	0.415	0.065
FLRT3	E2RKY7	0.451	0.445	0.360	0.459	0.456	0.028
FLVCR1	E2RSI2	0.305	0.352	0.368	0.258	0.175	0.072
FN1	F1P6H7	0.458	0.316	0.386	0.531	0.791	0.155
folh1	I4IY07	0.728	0.760	0.590	0.697	0.827	0.075
FOLR2	E2QXF0	0.683	0.701	0.687	0.646	0.679	0.016
FRRS1	E2RSW3	0.267	NaN	NaN	0.174	0.359	0.092
FUCA1	P48300	0.473	0.239	0.518	0.462	0.484	0.075
FZD1	F1PCN5	0.000	0.000	NaN	0.000	NaN	0.000
FZD5	E2QVP7	0.000	NaN	0.000	0.000	0.000	0.000
FZD6	F1P9V1	0.150	0.130	0.169	0.037	0.229	0.057
GARS	F1Q332	0.615	0.729	0.616	0.550	0.613	0.045
GLG1	F6V9R9	0.537	0.522	0.474	0.555	0.552	0.028
GLIPR1	F1PE15	0.112	0.122	0.101	0.207	0.103	0.031
GLS	E2RJ93	0.589	0.538	0.641	0.436	0.647	0.078
GM2A	F1P8U3	0.587	0.332	0.389	1.000	0.786	0.266
GNA13	F1PZW8	0.365	NaN	NaN	0.731	0.000	0.365
GNAI2	P38400	0.461	0.461	0.515	0.418	0.460	0.025
GNG12	J9P702	0.460	0.459	0.577	0.440	0.461	0.035
gns	Q32KH4	0.549	NaN	1.000	0.431	0.549	0.190
GPC1	F1PPA1	0.500	NaN	0.753	0.415	0.500	0.113
GPC4	F1PM35	0.083	0.000	0.000	0.257	0.165	0.106
GPNMB	E2QUR7	0.496	0.489	0.525	0.504	0.472	0.017
GPR107	F6USA6	0.223	NaN	NaN	0.000	0.445	0.223
GPR89A	E2R506	0.476	0.497	0.625	0.376	0.455	0.073
GPRC5A	F1PHN5	0.780	NaN	NaN	1.000	0.561	0.220
GRN	E2RHN1	0.561	0.465	0.624	0.609	0.513	0.064
HAVCR1	F1CME9	1.000	1.000	1.000	1.000	1.000	0.000
HEXB	K0J6C5	0.232	NaN	NaN	0.000	0.463	0.232
HFE	F1PX48	0.040	0.037	0.043	0.023	0.487	0.118
HM13	E2RB97	0.554	0.554	0.555	NaN	0.337	0.073
HMGCL	E2RFD0	0.500	0.503	0.503	0.477	0.497	0.008
HPN	E2RKP5	0.369	0.379	0.268	0.359	0.637	0.098
HSD17B4	E2R4T7	0.493	0.528	0.604	0.446	0.458	0.057
HSP90B1	F1P8N6	0.550	0.466	0.563	0.569	0.538	0.032
HSPG2	J9NRJ0	0.309	0.120	0.147	0.503	0.471	0.177
HTRA1	F1PU95	0.193	0.402	0.160	0.000	0.227	0.117
HYAL2	E2R560	0.501	0.499	0.567	0.454	0.503	0.029
HYOU1	E2RB31	0.498	0.528	0.638	0.468	0.394	0.076
ICAM1	P33729	0.862	0.853	0.851	0.870	0.941	0.027
IFI30	F1PK63	0.539	0.768	0.608	0.469	0.390	0.129
IFNGR1	F6XBS0	0.000	0.000	0.000	NaN	0.000	0.000
IGF1R	F1PXU6	0.315	0.334	0.307	0.323	0.303	0.012
IGFBP7	E2RNL2	0.000	0.000	0.000	0.000	NaN	0.000
IGSF11	F1PJL3	0.561	NaN	0.420	NaN	0.702	0.141

IGSF3	F1P9K1	0.458	0.465	0.459	0.458	0.410	0.014
IGSF5	E2RBD3	1.000	1.000	1.000	1.000	1.000	0.000
IGSF8	J9NWL5	0.409	NaN	0.000	0.468	0.409	0.156
IGSF9	F1PHY9	0.325	0.347	0.333	0.316	0.293	0.018
IL1R1	F1PSF0	0.815	NaN	1.000	0.815	0.779	0.074
IL1RAP	J9NU59	0.560	0.507	0.590	0.529	0.603	0.039
IL6ST	F1PTB2	0.702	0.489	NaN	0.702	0.950	0.154
ITGA1	F1PP33	0.330	0.365	0.325	0.307	0.336	0.017
ITGA2	E2REA9	0.532	0.605	0.570	0.485	0.495	0.049
ITGA3	F1Q439	0.250	0.283	0.240	0.230	0.259	0.018
ITGA6	E2RL88	0.287	0.311	0.287	0.287	0.272	0.010
ITGAV	F1P8Q0	0.429	0.460	0.446	0.412	0.401	0.023
ITGB1	E2RT60	0.346	0.382	0.352	0.319	0.341	0.018
ITGB5	J9P3C1	0.545	NaN	0.623	0.486	0.545	0.045
ITGB6	E2RBL9	0.381	0.390	0.387	0.362	0.375	0.010
ITGB8	F6X717	0.447	0.000	0.278	0.615	0.653	0.248
ITPR3	E2RMC0	0.473	0.372	0.445	0.525	0.502	0.053
KIAA1161	J9PAS5	0.076	0.087	0.000	0.076	NaN	0.029
KIRREL	F1Q051	0.026	0.000	0.000	0.052	0.128	0.045
KTN1	E2RG27	0.464	0.437	0.498	0.447	0.481	0.024
L1CAM	E2R2V6	0.578	0.635	0.622	0.522	0.533	0.050
LAMA3	E2RPP1	0.396	0.705	0.395	0.388	0.398	0.080
LAMB1	F1PCD8	0.422	0.456	0.238	0.447	0.397	0.067
LAMB3	F1PFM5	0.406	0.387	0.339	0.425	0.453	0.038
LAMC1	F1PHK9	0.296	0.071	0.085	0.506	0.549	0.225
LAMP1	F1Q260	0.474	0.513	0.597	0.409	0.435	0.067
LAMP2	E2RNJ1	0.090	1.000	0.000	0.000	0.181	0.295
LDLR	F1PML2	0.361	NaN	0.361	0.475	0.204	0.091
LGALS3BP	E2RKQ6	0.539	0.562	0.594	0.516	0.499	0.035
LGMN	E2QXF2	0.485	0.481	0.310	0.489	0.567	0.066
LHFPL2	J9P9A6	0.516	0.524	0.533	0.394	0.507	0.039
LIPH	F1PG57	0.231	0.168	0.253	0.210	0.324	0.050
LMAN2	P49256	0.543	0.549	0.536	0.559	0.380	0.048
LMF2	J9NVV3	0.518	0.533	0.549	0.475	0.503	0.026
LRP1	J9P315	0.239	0.197	0.161	0.281	0.299	0.056
LRPAP1	F1PAG7	0.852	NaN	NaN	1.000	0.705	0.148
LSAMP	F1PJN2	0.725	0.751	0.734	0.716	0.709	0.015
LSR	F1PKT2	0.130	0.115	0.096	0.148	0.145	0.020
LTBR	E2RG08	0.010	0.000	0.007	0.013	0.098	0.026
M6PR	E2R4C1	0.201	0.209	0.154	0.193	0.228	0.022
MAGT1	E2RRH2	0.546	0.526	0.566	0.196	0.576	0.105
MET	Q75ZY9	0.406	0.403	0.360	0.416	0.409	0.015
METTL7A	J9NTA1	0.454	0.446	0.449	0.498	0.460	0.016
MFGE8	F1PFZ5	0.731	0.710	0.673	0.778	0.752	0.037
MFSD6	E2RK97	0.044	0.000	0.000	0.307	0.087	0.099
MLEC	E2RD92	0.457	0.479	0.611	0.412	0.436	0.060
MME	F5C3N2	1.000	NaN	NaN	1.000	1.000	0.000
MMP14	F1PXM2	0.264	0.324	0.096	0.305	0.224	0.077
MMP15	F1P625	0.116	0.034	0.065	0.167	0.269	0.085
MPZL1	J9NSZ7	0.124	NaN	0.000	0.124	0.160	0.053
MRPL21	J9P2W1	0.000	0.000	0.000	NaN	0.437	0.146
MRPS35	E2R2D2	0.309	0.518	0.000	0.281	0.338	0.143
MSLN	J9NX38	0.834	0.977	0.881	0.654	0.787	0.104
MUC1	J9P6J1	0.901	0.894	0.880	0.907	0.931	0.016
MUC20	F1PJ84	0.929	0.840	0.858	1.000	1.000	0.076
NCLN	E2QY93	0.413	0.513	0.604	0.313	0.000	0.201
NCSTN	M1VEJ1	0.267	0.249	0.000	0.285	0.394	0.107
NDUFA10	E2RIG0	0.489	0.500	0.418	0.477	0.598	0.051
NEO1	J9P2T1	0.335	0.101	0.333	0.338	0.433	0.084
NNT	F1PLG2	0.467	0.547	0.491	0.295	0.443	0.075
NOTCH2	F1PPP5	0.226	0.000	0.214	0.298	0.238	0.081
NPC1L1	A0MJA4	0.929	0.934	0.931	0.927	0.923	0.004
NPC2	Q28895	0.375	0.375	0.310	NaN	0.679	0.123
NPDC1	F6XGX1	0.000	NaN	0.000	NaN	0.000	0.000
NPTN	F1PDQ0	0.040	0.037	0.022	0.069	0.044	0.014
NTN4	E2QZY0	0.180	0.000	NaN	0.359	NaN	0.180

NUP155	F6XTD9	0.411	0.256	0.437	0.384	0.460	0.064
NUP210	F1PSK9	0.507	0.490	0.636	0.507	NaN	0.049
OCLN	E2R3X4	0.156	0.000	0.152	0.160	0.169	0.044
OSMR	E2QWS7	0.631	0.593	0.628	0.634	0.908	0.080
P4HA1	E2RLB8	0.591	0.606	0.646	0.575	0.504	0.043
P4HA2	F1PTP6	0.552	0.649	NaN	NaN	0.455	0.097
PCCB	F1PTU4	0.482	0.533	0.508	0.406	0.457	0.045
PCDH1	E2QX93	0.219	0.191	0.151	0.247	0.248	0.038
PCYOX1	E2R1N7	0.388	0.243	1.000	0.534	0.203	0.272
PGAP1	E2R8V9	0.493	0.502	NaN	0.493	0.488	0.005
PIGN	F1PVD7	0.689	1.000	NaN	NaN	0.379	0.311
PIGO	E2RKS8	0.494	0.557	0.516	0.473	0.436	0.041
PIGR	F6Y6T8	0.411	0.369	0.316	0.452	0.496	0.066
PLAUR	E2RGN0	0.804	0.795	0.666	0.814	0.831	0.046
PLD3	E2QVP0	0.350	NaN	0.561	0.350	0.339	0.074
PLOD2	F1PZL5	0.448	0.407	0.337	0.857	0.489	0.150
PLOD3	E2RTL4	0.389	NaN	0.000	0.389	1.000	0.333
PLXNA1	F1P6G0	0.216	0.141	0.178	0.255	0.392	0.082
PLXNA2	F1PHZ1	0.421	0.441	0.447	0.400	0.327	0.040
PLXNB1	E2RM56	0.213	0.215	0.215	0.209	0.212	0.003
PLXNB2	F1P9S5	0.307	0.320	0.309	0.292	0.304	0.008
PLXNB3	F1PXM6	0.000	0.000	0.209	NaN	0.000	0.070
PMPCA	F1PF09	0.488	0.513	0.463	0.407	0.632	0.069
PNPLA6	E2RIR2	0.497	0.379	0.504	0.497	NaN	0.042
PODXL	F1PID4	0.928	0.917	0.918	0.937	0.950	0.013
PON2	F1PSG5	0.483	0.403	0.513	0.454	0.512	0.042
PPIB	F1PLV2	0.703	0.555	0.754	0.741	0.666	0.069
PRCP	F1PWK3	0.264	0.000	NaN	NaN	0.528	0.264
PRKCSH	E2RKK6	0.795	0.590	1.000	1.000	0.579	0.208
PrP	O46593	0.697	0.698	0.707	0.656	0.697	0.013
PTGER2	F1P776	0.000	0.000	0.000	NaN	NaN	0.000
PTGFRN	F1PR26	0.346	0.379	0.370	0.322	0.322	0.026
PTK7	E2RMT2	0.433	0.462	0.423	0.395	0.443	0.022
PTPRA	E2R5J7	0.000	NaN	0.000	NaN	0.000	0.000
PTPRD	F1PH17	0.492	0.441	0.413	0.564	0.542	0.063
PTPRF	E2RE80	0.364	0.337	0.334	0.402	0.391	0.030
PTPRG	F1PWC1	0.272	0.184	0.259	0.288	0.285	0.033
PTPRJ	F1P983	0.941	0.971	0.867	0.931	0.951	0.031
PTPRM	F1PUP2	0.422	0.421	0.431	0.403	0.423	0.008
PTPRO	F1PWH3	1.000	0.744	1.000	1.000	1.000	0.064
PTPRS	E2R6Z2	0.154	0.078	0.065	0.230	0.310	0.099
PVR	F1PVS7	0.362	0.390	0.384	0.340	0.309	0.031
RAB10	F2Z4P9	0.464	0.553	0.489	0.410	0.440	0.048
RAB13	F1PTE3	0.459	0.476	0.000	0.493	0.443	0.131
RAB25	E2RQ15	0.500	0.533	0.581	0.466	0.437	0.053
RAB5A	P18066	0.456	0.404	0.499	0.443	0.468	0.030
RAB5C	P51147	0.470	0.375	0.467	0.541	0.473	0.043
RAB8A	P61007	0.460	0.502	0.512	0.407	0.418	0.047
RAB9A	P24408	0.525	NaN	0.381	0.598	0.525	0.072
RAC1	P62999	0.482	0.474	0.490	0.389	0.536	0.041
RDH14	J9NWS8	0.485	0.381	0.511	0.459	0.594	0.066
RELL1	E2QUZ0	0.129	0.149	0.113	0.027	0.146	0.039
RPN1	E2RQ08	0.515	0.538	0.563	0.478	0.493	0.033
RPSA	J9JHJ2	0.421	0.422	0.420	0.412	0.520	0.027
RTN4	E2R925	0.582	0.565	0.599	0.624	0.553	0.026
SCARB1	F1PQT1	1.000	1.000	1.000	NaN	NaN	0.000
SCPEP1	J9NRZ7	0.536	0.618	0.453	1.000	0.096	0.267
SDC4	J9NZ79	0.082	0.044	0.041	0.126	0.121	0.040
SDHB	Q0QEY4	0.492	0.543	0.682	0.362	0.441	0.106
SEC61A1	J9NXA9	0.487	0.581	0.492	0.479	0.482	0.028
SEMA4B	F1P9X6	0.311	0.203	0.230	0.411	0.392	0.092
SEMA4C	E2RHV1	0.356	0.271	0.195	0.440	0.453	0.107
SEMA4D	F1PSB1	0.381	0.315	0.299	0.447	0.485	0.079
SEMA4G	E2RP18	0.104	0.000	0.038	0.199	0.170	0.083
SERPINH1	E2RHY7	0.497	0.481	0.544	0.485	0.508	0.022
SFTPD	E2QWR0	0.683	NaN	0.000	0.683	1.000	0.333

SIAE	F1PXN3	0.531	0.454	0.609	NaN	NaN	0.077
SIGIRR	F1PY07	0.083	0.000	0.000	0.166	0.172	0.085
SLC12A2	F1P9W5	0.167	0.107	0.130	0.203	0.281	0.062
SLC12A6	F1Q2J3	0.120	0.127	0.416	0.000	0.113	0.107
SLC12A7	F1PKH1	0.220	0.000	0.354	0.220	NaN	0.118
SLC15A4	F1PWH8	0.463	0.619	NaN	0.463	0.444	0.058
SLC16A1	F1PCQ3	0.195	0.218	0.173	0.180	0.209	0.018
SLC16A3	J9NVM8	0.363	0.405	0.456	0.249	0.322	0.072
SLC1A4	F1Q0H9	0.000	0.000	0.000	0.000	0.000	0.000
SLC1A5	K7ZSN9	0.053	0.000	0.059	0.047	0.307	0.080
SLC22A2	F1Q431	0.344	0.426	NaN	0.261	NaN	0.082
SLC27A4	F1PAM6	0.515	0.514	0.516	0.471	1.000	0.132
SLC2A1	F1PWN2	0.468	0.506	0.553	0.346	0.430	0.071
SLC31A1	J9P9D2	0.302	0.000	0.405	0.308	0.295	0.104
SLC33A1	E2RTD2	0.380	0.356	0.583	0.404	0.335	0.074
SLC35F6	J9P2U5	0.537	0.536	0.538	0.639	0.444	0.049
SLC37A4	F1PPG9	0.527	0.588	0.427	0.544	0.509	0.049
SLC39A4	F1PC23	0.908	0.854	0.901	0.929	0.914	0.022
SLC39A6	E2RMA6	0.139	0.177	0.015	0.285	0.101	0.086
SLC39A7	Q5TJF6	0.435	0.501	0.000	0.435	NaN	0.167
SLC3A2	F1PRC5	0.202	0.233	0.219	0.185	0.171	0.024
SLC44A1	E2R949	0.133	0.134	0.099	0.131	0.139	0.011
SLC44A2	J9P0D9	0.264	0.281	0.219	0.246	0.352	0.042
SLC44A4	F1P9U5	0.947	0.880	0.954	0.950	0.944	0.020
SLC4A7	J9PAF7	0.063	0.060	0.082	0.042	0.066	0.012
SLC52A3	E2RM06	1.000	NaN	1.000	1.000	1.000	0.000
SLC5A6	E2R9T1	0.763	0.911	0.726	0.801	0.689	0.074
SLC6A6	Q00589	0.226	0.296	0.000	0.175	0.276	0.099
SLC7A5	F1PH98	0.183	0.264	0.172	0.186	0.180	0.025
SLC9A1	F1PID1	0.079	0.121	0.066	0.054	0.091	0.023
SLIT3	F1Q3G2	0.381	0.197	0.128	0.564	0.567	0.202
SLITRK4	F1Q4K7	0.417	0.390	0.439	0.402	0.431	0.020
SPINT1	F1PQ47	0.354	0.334	0.328	0.384	0.374	0.024
SPINT2	F1P984	0.225	0.173	0.201	0.249	0.251	0.032
SSR1	P16967	0.512	0.478	0.546	0.139	0.713	0.160
SSR4	E2QY54	0.544	0.589	0.635	0.452	0.500	0.068
ST14	F1PF63	0.206	0.187	0.173	0.225	0.311	0.044
STEAP3	F1P8R0	0.288	0.240	0.277	0.298	0.416	0.049
STT3A	F1PJP5	0.508	0.510	0.548	0.480	0.507	0.017
STT3B	E2RG47	0.476	0.548	0.541	0.299	0.412	0.094
SUCLG1	E2R0Y5	0.444	0.418	0.544	0.313	0.469	0.071
SUOX	F1PVD4	0.500	0.275	0.441	1.000	0.560	0.211
SYNGR2	F1PZ37	1.000	1.000	1.000	NaN	0.000	0.333
SYPL1	E2RC10	0.324	0.327	0.337	0.242	0.320	0.025
TAPBP	Q5TJE4	0.535	0.598	0.594	0.477	0.472	0.061
TBL2	E2RP67	0.478	0.532	0.566	0.346	0.424	0.082
TENM4	F1PFD6	0.502	0.655	0.543	0.461	0.439	0.074
TEX264	E2RAC8	0.464	NaN	0.552	NaN	0.376	0.088
TF	F6V1W9	0.438	0.437	0.506	0.438	0.437	0.018
TGFBR3	F1PIG0	0.310	0.310	0.311	0.299	0.352	0.014
TIMM21	E2R117	0.496	NaN	0.627	0.000	0.496	0.209
TINAGL1	E2QXH3	0.249	0.000	0.187	0.330	0.310	0.113
TM2D3	F6UUM9	0.234	0.606	NaN	0.234	0.177	0.143
TM7SF2	E2RS95	0.512	0.565	0.575	0.444	0.460	0.059
TM9SF2	E2R5Z5	0.416	0.513	0.427	0.232	0.404	0.076
TM9SF3	E2RSD0	0.441	0.571	0.474	0.290	0.407	0.087
TMED10	E2QYL0	0.537	0.581	0.698	0.493	0.464	0.080
TMED9	F6XD36	0.495	0.609	0.529	0.458	0.461	0.055
TMEM109	J9P616	0.605	0.547	0.662	0.793	0.471	0.109
TMEM2	F1PH14	0.353	0.323	0.329	0.399	0.378	0.031
TMEM30A	E2R2Z8	0.349	0.437	0.418	0.067	0.280	0.127
TMPRSS11E	F1Q3A2	0.297	0.319	0.303	0.270	0.290	0.015
TMX1	E2RK67	0.533	0.590	0.560	0.434	0.506	0.052
TMX3	F1PIX5	0.516	0.432	NaN	0.516	0.681	0.083
TPBG	E2QXI6	0.293	0.230	0.287	0.336	0.298	0.029
TRPA1	F1Q2M0	0.000	0.000	0.414	0.000	0.000	0.104

TSPAN1	J9NS67	0.645	0.537	NaN	0.645	1.000	0.154
TSPAN14	E2QZH1	0.228	0.113	0.214	0.251	0.241	0.042
TSPAN15	E2RS68	0.128	0.054	0.127	0.129	0.167	0.029
TSPAN33	F1PEJ5	0.034	0.000	0.000	0.067	0.149	0.054
TSPAN6	F1PMP0	0.000	0.000	0.000	0.000	NaN	0.000
TSPAN8	E2QSC8	0.220	0.236	0.240	0.190	0.204	0.020
TTYH3	F1P9A7	0.317	0.335	0.134	0.302	0.332	0.058
UNC93B1	F1PXK2	0.414	0.549	0.000	0.348	0.480	0.170
UPK3B	E2R4D0	0.838	0.830	0.861	0.846	0.829	0.012
VASN	E2RRN8	0.121	0.051	0.077	0.165	0.165	0.051
VASP	P50551	0.524	0.452	0.524	0.524	0.548	0.024
VCAN	J9P884	0.197	0.053	0.138	0.255	0.279	0.086
VDAC2	E2R948	0.476	0.576	0.541	0.344	0.412	0.091
VLDLR	F1P787	0.100	0.119	0.082	0.039	0.300	0.074
VNN1	Q9TSX8	0.750	0.736	0.780	0.738	0.762	0.017
VSIG10	J9P1R7	0.176	0.241	0.000	1.000	0.111	0.282
WFDC2	F1PDT8	0.486	0.566	0.486	0.358	NaN	0.069
